# Global evidence of positive impacts of freshwater biodiversity on fishery yields

**DOI:** 10.1111/geb.12435

**Published:** 2016-02-01

**Authors:** Emma Grace Elizabeth Brooks, Robert Alan Holland, William Robert Thomas Darwall, Felix Eigenbrod, Derek Tittensor

**Affiliations:** ^1^ Centre for Biological SciencesUniversity of SouthamptonSouthamptonSO17 1BJUK; ^2^ Global Species ProgrammeIUCN (International Union for Conservation of Nature)CambridgeCB3 0DLUK

**Keywords:** Biodiversity, ecosystem services, fisheries, freshwater, productivity, resilience

## Abstract

**Aim:**

An often‐invoked benefit of high biodiversity is the provision of ecosystem services. However, evidence for this is largely based on data from small‐scale experimental studies of relationships between biodiversity and ecosystem function that may have little relevance to real‐world systems. Here, large‐scale biodiversity datasets are used to test the relationship between the yield of inland capture fisheries and species richness from 100 countries.

**Location:**

Inland waters of Africa, Europe and parts of Asia.

**Methods:**

A multimodel inference approach was used to assess inland fishery yields at the country level against species richness, waterside human population, area, elevation and various climatic variables, to determine the relative importance of species richness to fisheries yields compared with other major large‐scale drivers. Secondly, the mean decadal variation in fishery yields at the country level for 1981–2010 was regressed against species richness to assess if greater diversity reduces the variability in yields over time.

**Results:**

Despite a widespread reliance on targeting just a few species of fish, freshwater fish species richness is highly correlated with yield (*R* ^2^ = 0.55) and remains an important and statistically significant predictor of yield once other macroecological drivers are controlled for. Freshwater richness also has a significant negative relationship with variability of yield over time in Africa (*R* ^2^ = 0.16) but no effect in Europe.

**Main conclusions:**

The management of inland waters should incorporate the protection of freshwater biodiversity, particularly in countries with the highest‐yielding inland fisheries as these also tend to have high freshwater biodiversity. As these results suggest a link between biodiversity and stable, high‐yielding fisheries, an important win–win outcome may be possible for food security and conservation of freshwater ecosystems. However, findings also highlight the urgent need for more data to fully understand and monitor the contribution of biodiversity to inland fisheries globally.

## Introduction

The degree to which species diversity underpins ecosystem functioning, and ultimately ecosystem services, is a central question in ecology with significant implications for policy and conservation (Mace *et al*., 2012). It is now well established that biodiversity frequently has a positive effect on ecosystem functioning (Balvanera *et al*., 2006; Duffy, 2009; Cardinale *et al*., 2011). However, not all studies support this conclusion (Hooper *et al*., 2005). Observational studies appear to contradict results from experimental studies, and vary in clarity and the direction of any relationship (Naeem, 2002). It is therefore possible that biodiversity–ecosystem functioning experiments may not be indicative of realized differences in ecosystem functioning in natural systems. This disconnect may in some cases be due to differences in scale and system (Duffy, 2009), and there is a disparity between the small scales at which these experiments have been performed and the scale at which management and conservation decisions are made (Cardinale *et al*., 2012).

The use of real‐world datasets bypasses some of these issues, yet currently there are only a handful of sufficiently large‐scale studies with which to examine the effect of biodiversity on ecosystem function in natural ecosystems. To date global studies have examined marine (Worm *et al*., 2006) and botanical (Maestre *et al*., 2012) systems. To understand whether there is a generalizable relationship between ecosystem function and biodiversity or whether a more idiosyncratic relationship exists there is a need to examine evidence across a range of scales, systems and taxa. Moreover, more work is needed to look beyond questions of generalized functionality and productivity and consider direct links to human wellbeing. Such work is important because there is a much poorer understanding of the links between biodiversity and the final ecosystem goods that actually confer benefits to humans (Mace *et al*., 2012).

The question of the extent to which biodiversity underpins ecosystem services is relevant to a range of systems, but has perhaps the greatest policy relevance in terms of the provisioning services that underpin food security. Studies of the relationship between ecosystem functioning and biodiversity show that increased species richness (SR) may provide (1) a buffering effect in fluctuations of productivity and/or (2) an overall performance‐enhancing effect (Yachi & Loreau, 1999). However, as these conclusions have been largely drawn from plant community experiments these mechanisms may have limited generality across systems (Pinto *et al*., 2013). Although current focus in this area is on examining the impacts of biodiversity within human agricultural systems (e.g. as reviewed in Power, 2010), people also rely on natural habitats for the provision of food.

At least 2 billion people depend directly on inland freshwaters for the provision of food (Richter *et al*., 2010), and in many parts of the world inland waters are often the primary source of protein and micronutrients (Béné *et al*., 2007; Dugan *et al*., 2010). In 2010, global inland capture fisheries yielded over 11 million tonnes, with inland aquaculture yielding up to four times that amount (FAO, 2012). Globally there are hundreds, if not thousands, of freshwater species that contribute to food security, yet the relationship between species diversity and yield remains poorly understood in freshwater systems (Balmford *et al*., 2008). Recent research suggests a performance‐enhancing effect (Greene *et al*., 2010; Carey & Wahl, 2011) and a buffering effect (Greene *et al*., 2010; Franssen *et al*., 2011) of biodiversity on yield associated with freshwater fish communities, although it is unclear how such results transfer to natural freshwater systems at larger scales (Carey & Wahl, 2011). Different species can make a disproportionate contribution to ecosystem functions (McIntyre *et al*., 2007), but in practice most fisheries concentrate on maximizing biomass – which is highly affected by such factors as phosphorus levels and macrobenthos biomass in freshwater systems (Hanson & Leggett, 1982) – and have little interest in harvesting a diversity of species. As a consequence, there is no degree of certainty that higher freshwater biodiversity is linked to enhanced livelihoods and increased human wellbeing. Indeed most fishery managers would prefer the ease of managing a fishery based on fewer species for which stock assessment tools aiming at maximum sustainable yield are more easily applied. Therefore greater comprehension is needed of how the relationship between biodiversity and ecosystem functioning can influence our understanding of the implications of freshwater biodiversity loss, and contribute to defining management objectives for inland freshwater systems (Dudgeon, 2010).

Beyond food security, understanding the degree to which biodiversity underpins freshwater fisheries has particular policy relevance because freshwater systems are of major importance for the conservation of biodiversity. Freshwater habitats are disproportionately species rich given that they cover only 0.8% of the Earth's surface; 10% of species described to date and as many as a third of all vertebrates are confined to freshwater habitats (Dudgeon *et al*., 2006). Freshwater systems are highly threatened, with many freshwater taxonomic groups facing a significantly higher extinction risk than terrestrial groups (Darwall *et al*., 2008). As a result, if freshwater biodiversity is shown to generally underpin inland fisheries, the food security implications of this relationship would provide a powerful additional argument to conserve freshwater systems and the biodiversity contained within them above and beyond purely conservation objectives.

Here, datasets from the Food and Agriculture Organization of the United Nations (FAO) and the International Union for Conservation of Nature (IUCN) covering 100 countries are used to provide the first large‐scale test of the hypotheses that high freshwater biodiversity has a positive effect on (1) fishery yields and (2) variability of yield over time. As ecosystem function is a result of more than just the target species (Hensel & Silliman, 2013), the analyses were conducted using fish SR and then repeated to include additional freshwater faunal groups, namely molluscs, odonates and decapods (see Appendix S4 in the Supporting Information).

## Methods

All analyses, unless otherwise specified, were conducted in R, version 3.0.2 (R Core Team, 2014).

### Freshwater biodiversity and yield

**Figure 1:**
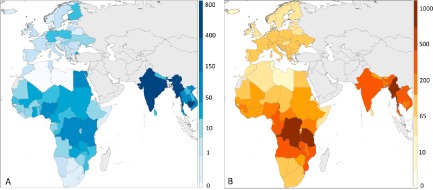
Data included within the study. (a) FAO inland water capture fisheries yield per country (thousands of tonnes) (axis quarter‐root transformed). (b) Freshwater fish species richness per country (axis cube‐root transformed).

The relationship between yields of inland capture fisheries and biodiversity was examined using comprehensive datasets from IUCN (2012) and FAO (2011) along with other macroecological drivers (see Appendix 1 for all data sources). Biodiversity analysis was based on species native range maps of 9075 freshwater species from the IUCN Red List of threatened species (IUCN, 2012), including 5203 species of fish, 1790 molluscs, 1329 odonates and 753 decapods. Range maps are compiled by experts in accordance with the IUCN Red List guidelines (available at http://www.iucnredlist.org) and derived from a combination of known and expected species localities. The IUCN spatial dataset is the most comprehensive continental‐scale dataset available on the distribution of all known freshwater taxa from these groups mapped to the river/lake subcatchment scale. SR per country was calculated in ArcMap 10 (ESRI, Redlands, CA, USA) using the range maps from IUCN Red List assessments for fish alone and then all available freshwater taxa. Countries for inclusion in the analyses were restricted to those that have a complete suite of species range maps for the taxonomic groups considered and have been comprehensively assessed by IUCN, namely Africa, Europe and parts of Asia (see Fig. 1b).

The FAO capture database FishStatJ is the most authoritative assessment of the status of world inland fisheries and reports national annual yield data since 1950 that can be filtered by country, taxonomy, fishing area and yield measures (FAO, 2011).

### Macroecological and human drivers of fisheries yield

Fisheries yield is a product of biophysical drivers and human effort. As such, quantifying the effect of biodiversity (SR) on mean yield (t) was achieved by first building a predictive model of all putative large‐scale drivers of fishery yield and then determining the relative importance of SR compared with the other drivers – area of surface water, fishing effort, productivity and elevation. Mean yield (t) was calculated at the country level from data for 2001–10 to best reflect the time period of the IUCN species assessments (conducted from 2004 to 2010) (Fig. 1a). The sum of the surface area of inland waters (km^2^) per country was extracted from the Global Lakes and Wetlands Database (GLWD) (Lehner & Döll, 2004) to account for available habitat for freshwater species. As no comprehensive data exist on per unit effort of fisheries or number of fishers at the country level, the human population within 10 km of inland waters for each country (hereafter the waterside population) was used as a proxy for fisheries effort. Population was extracted from a raster layer of the 2000 global rural population reported by FAO at 5‐arcmin resolution (Salvatorre *et al*., 2005), and a range of buffers around each of the waterbodies included in the GLWD were applied using ArcMap 10. Waterside population was validated against primary data on fishing effort available for 28 African lakes (see Appendix S1), with the 10‐km buffer yielding the strongest relationship (*r* = 0.75, *n* = 28, *P* < 0.001). Yield was not standardized by fishing effort or area (*sensu* Kantoussan *et al*., 2014). This is because both fishing effort (waterside population) and area affect the yield relationship at the country level; considering both as predictor variables accounts for this shared effect. A more in‐depth discussion of this issue is included in Appendix S1.

Productivity, or energy available within the system (which is highly correlated with climate at the continental scale; Hawkins *et al*., 2003), is a major driver of global freshwater biodiversity patterns (Tisseuil *et al*., 2012). As no spatial data currently exist on global freshwater productivity, this factor was controlled for by using the principal components from a principal components analysis (PCA) carried out on 19 spatial climatic data layers including mean and seasonality for temperature and precipitation variables (see Appendix S2). A broken stick stopping model selected the first two principal components for inclusion in the model; these together account for 79.8% of climate variation. Use of PCA in this way reduces multidimensionality and eliminates collinearity between variables, and is an established approach for controlling for productivity across latitudes in continental‐scale analyses (Hawkins *et al*., 2003; Tisseuil *et al*., 2012). Finally, mean elevation (m) was collated for each country.

### Relationship between freshwater biodiversity and yield

All possible linear regression models were built using mean yield (t) as the dependent variable, with fish SR, waterside population, climatic PCA components, inland water surface area (km^2^) and mean elevation (m) as explanatory variables. Testing the residuals of the models using Moran's *I* standard deviate test showed that there was spatial autocorrelation. Therefore a multimodel inference approach using simultaneous autoregressive spatial model (SAR) methods was conducted following Maestre *et al*. (2012). The type of SAR used was the spatial simultaneous autoregressive error model (SAR_err_) method, as this is robust to the type of spatial autocorrelation that is present in the data (Kissling & Carl, 2008), calculated within the spdep package in R. Spatial autocorrelation is accounted for by the inclusion of a spatial weighting matrix calculated based on distances between centroid points of countries. This spatial weighting matrix represents an additional term within the SAR model that describes relatedness between individual samples (countries) caused by spatial structure that is not fully accounted for by the other model parameters (Dormann *et al*., 2007). Where necessary, Box–Cox transformations were used to normalize the distribution of the residuals, equalize the variance and improve the fit of the models (Osborne, 2010). The full SAR models are presented with the results tables (Table 1 and Table S3 in Appendix S6).

**Table 1:** Simultaneous autoregressive spatial models of country‐level inland water fisheries yield (t) (quarter‐root transformed). (a) Two best fitting models. (b) The same models repeated excluding species richness as a variable. Shaded cells indicate which of the biodiversity, climatic and geographical variables were included in the model

(a)									
SR	*P*	*C*1	*C*2	*A*	*E*	Pseudo *R* ^2^	AIC_c_	ΔAIC_c_	*W_i_*
						0.76	545.50	0	0.39
						0.76	547.35	1.85	0.15

(b)									
SR	*P*	*C*1	*C*2	*A*	*E*	Pseudo *R* ^2^	AIC_c_	ΔAIC_c_	*W_i_*
						0.74	552.31	6.81	0.01
						0.74	552.72	7.22	0.01

SR, species richness of fishes (cubic‐root transformed); *P*, human population living within 10 km of inland waterbodies (quarter‐root transformed); *C*1, first principal component of climatic variables; *C*2, second principal component of climatic variables; *A*, inland water area in km^2^ (quarter‐root transformed); *E*, mean elevation (m) (cubic‐root transformed); ΔAIC_c_, difference between the AIC_c_ of each model and that of the best model; *W_i_*, Akaike weight. Full model as calculated in the spatial simultaneous autoregressive error model: $\sqrt[{4}]{\text{Yield}}=\sqrt[{3}]{\text{SR}}+\sqrt[{4}]{P}+C1+C2+\sqrt[{4}]{A}+\sqrt[{3}]{E}$.

From all possible models, minimized second‐order Akaike information criteria corrected for small sample size (AIC_c_) were used to select the best fitting models. The AIC_c_ of all models selected that included SR as a predictor were compared with those of the same models not including SR. Where the AIC_c_ of models differs by less than 2, the models are considered to be indistinguishable (Burnham & Anderson, 2002). The Akaike weights of each model were calculated based on the ΔAIC_c_, i.e. the difference between the AIC_c_ of each model and that of the best model (Burnham & Anderson, 2002), and therefore a set was created from all models where ΔAIC_c_ was different by less than two from the best model, hereafter known as the top model set. Multimodel‐averaged parameter estimates of the analysis were calculated using the top model set. The relative importance of each predictor variable was calculated as the sum of the Akaike weights of all models that included the predictor of interest (Burnham & Anderson, 2002). Commonality analysis was then conducted to determine the unique, common and total effects of each of the variables within each of the top model sets (Nimon & Reio, 2011). The variance inflation factor (VIF) was calculated for the top models to check for collinearity between predictor variables.

### Relationship between freshwater biodiversity and variability of yield

The coefficient of variation (CV) is a measure commonly used to quantify variation within a system (e.g. Pinto *et al*., 2013). The CV of yield (t) was calculated for each country for three decadal increments from the years 1981–2010, and the mean CV was compared with linear regression to fish SR per country. Records prior to 1981 were excluded due to the higher chance of inaccuracies and extrapolated figures with older data (Garibaldi, 2012). As before, Box–Cox methodology was used to determine the most appropriate transformation to ensure that data fitted modelling assumptions. The analysis was repeated for country data subset by continent for comprehensive datasets (Africa and Europe).

When examining the link between biodiversity and variation in fisheries yield, CV would not differentiate between a yield which is steadily increasing or decreasing and one which is unstable but fluctuating in similar increments around the mean (see Fig. S3 in Appendix S5). Therefore a variation measure was adapted to consider differences of year on year yield (see Appendix S3) and calculated alongside CV for comparison.

## Results

### Freshwater biodiversity and yield

**Figure 2:**
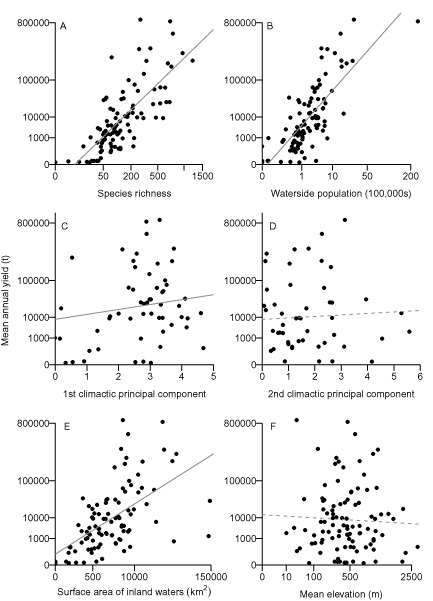
Relationship between inland water capture fisheries mean annual yield (t) (axes quarter‐root transformed) and model predictor variables at the country level (*n* = 100): axes for fish species richness and mean elevation (m) are cubic‐root transformed. Details of the climatic principal components are given in Table S1 in Appendix S6. Solid lines show *P* < 0.05, dashed lines are non‐significant.

Considered in isolation, there is a strong positive relationship between fish SR and mean annual yield (t) (*R* ^2^ = 0.55, *F* = 122.6, *P* < 0.001). The relationship between mean annual yield (t) and each of the macroecological drivers used in the models is shown in Fig. 2.

Fish SR is an important predictor of overall fisheries yield in the global SAR models that include it and the other major macroecological drivers of yield. These global models – which explain most of the variation in fisheries yield (*R^2^* = 0.76; Table 1) – shows that all the variables considered here are important for predicting fisheries yield with the exception of the second principal component of the climatic variables. SR is present in both of the best‐fitting models (Table 1a) among the 64 possible models. These top models have the smallest AICc and fewest variables for models with comparable AICc. The residual difference per country for the full model is shown in Fig. S2 in Appendix S5.

Excluding SR from the models resulted in a reduction in mean adjusted *R* ^2^ of 0.02 (Table 1b). SR made a contribution of between 0.03 and 0.05 in unique effects for each of the top models (Table 2), while area contributed 0.03, and waterside population contributed between 0.06 and 0.07 in unique effects for each model. When shared variation was also considered, SR contributed a total effect of 0.55 (accounting for 72% of pseudo *R* ^2^), far greater than the other variables except for waterside population, which contributed 0.60 (79%) (Table 2). The overall contributions of each of the variables within the models are shown in Table 2 and Table S2 in Appendix S6. With the VIFs between predictor variables in all of the best models being well below 10 there was no suggestion of undue collinearity between variables (Table 3).

**Table 2:** Commonality coefficients of the top model set of simultaneous autoregressive spatial models of country‐level inland water fisheries yield (t). Abbreviations as in Table 1

Model	Variable (*x*)	Unique	Common	Total	% of *R* ^2^
1	SR	0.05	0.50	0.55	72
	P	0.06	0.54	0.60	79
	C1	0.01	0.23	0.24	32
	A	0.03	0.32	0.34	45
	E	0.01	−0.01	0.002	0.3
2	SR	0.03	0.52	0.55	72
	P	0.07	0.53	0.60	79
	C1	0.01	0.23	0.24	32
	C2	0.003	0.007	0.01	1
	A	0.03	0.32	0.34	45
	E	0.01	−0.001	0.002	0.3

Unique, unique effect of *x*; Common, sum of the common effects of *x*; Total, Unique + Common; % of *R* ^2^ = *Total*/*Adj*. *R* ^2^.

**Table 3:** Variance inflation factors of predictor variables of top model set of simultaneous autoregressive spatial models of country‐level inland water fisheries yield (t). Shaded cells indicate which of the biodiversity, climatic and geographical variables were included in the model. Abbreviations as in Table 1

SR	*P*	*C*1	*C*2	*A*	*E*
2.48	2.19	1.81		1.86	1.13
3.16	2.36	1.89	1.29	1.90	1.15

Finally, river fisheries are known to be as much as three times more productive than lakes (Randall *et al*., 1995) and therefore as a type of sensitivity analysis the models were repeated with river area weighted higher than lake area. This made no difference to the relative importance of fish SR or the total effects of fish SR upon the models, and is therefore not considered further in this study.

### Freshwater biodiversity and yield variability

**Figure 3:**
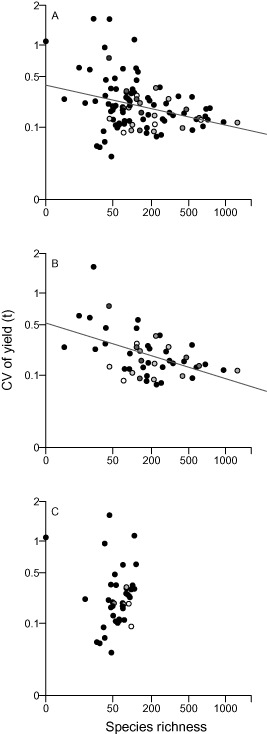
Relationship between fish species richness and mean coefficient of variation of yield (t) (both axes cubic‐root transformed). (a) All countries within the boundaries of this study (*n* = 100). (b) African countries (*n* = 48). (c) European countries (*n* = 41). The proportion of FAO yield data per country that has been estimated or extrapolated by FAO is graded from white (all years estimated) to black (all actual data).

Based on data for all countries included in this study there is no significant relationship between SR and CV of fisheries yield (t) (*R* ^2^ = 0.02, *F* = 4.14, *P* = 0.07; Fig. 3a). However, independent examination of continent‐scale data found a significant negative relationship between SR and CV when only African country data are examined (*R* ^2^ = 0.16, *F* = 9.81, *P* = 0.003; Fig. 3b); this is not present in European data (*R* ^2^ = −0.02, *F* = 0.22, *P* = 0.65, Fig. 3c). When variability was analysed using an adapted metric which examines year‐to‐year differences the negative relationship for all countries is significant (Table S5 in Appendix S6). At the continental scale, relationships are similar to those found using CV, although they are slightly weaker for the African data.

## Discussion

This work provides the first large‐scale analysis of the relationship between freshwater biodiversity and inland fisheries. In showing that there is positive effect of freshwater biodiversity on fishery yield at the global scale, these results extend the growing body of work that shows a positive effect of biodiversity on increased productivity (e.g. see reviews in Cardinale *et al*., 2011, 2012) by examining a final ecosystem good using large‐scale, real‐world data. Countries with higher freshwater fish SR report a higher mean yield – a finding that mirrors smaller‐scale work on freshwater fish in mesocosms (Carey & Wahl, 2011) and reservoirs in the American Midwest (Carlander, 1955) as well as on marine fisheries (Worm *et al*., 2006). Importantly, this finding holds after accounting for other macroecological and human drivers. Given the scale of the analysis and the number of covariates considered it is unsurprising that the independent effect of fish SR on yield is small (3–5%), and the effect is comparable to similar studies that have drawn analogous conclusions in different systems (e.g. Maestre *et al*., 2012).

Previous theoretical and empirical studies have suggested that there are both positive and negative relationships between biomass in fish communities and biodiversity (Hugueny *et al*., 2010). The two most prominent mechanisms generally responsible for positive relationships between yield and biodiversity are (1) the sampling effect, where dominant species increase productivity, and (2) the complementarity effect, where productivity is higher than would be suggested by consideration of individual species alone due to niche partitioning and facilitation (Loreau *et al*., 2001). Negative relationships between biodiversity and yield can also occur if one species is able to exploit a limiting resource to such an extent that it is less available to other species (density compensation; MacArthur *et al*., 1972).

The positive relationship between mean yield and SR revealed in the current analysis provides evidence for either a sampling or a complementarity effect. Data on the proportional contribution of species to total yield from the FAO (Table S6 in Appendix S6) indicate that for many countries over 50% of total yield is attributed to fewer than five species. This might suggest that the sampling effect is acting as a mechanism causing performance enhancement. If this was the sole mechanism then it cannot be concluded that biodiversity per se is responsible (Loreau *et al*., 2001) for the relationship described in the current study. However, inland fisheries are not entirely dominated by a handful of species in all countries (Table S6 in Appendix S6), and socioeconomic factors (e.g. targeted fishing of the most economically valuable species) rather than ecological community structure could contribute to the dominance of a few species in catch statistics. As such, complementarity effects of biodiversity are still possible, even for a fishery whose yields are largely dependent on a few exploited species. Indeed, previous studies have demonstrated that complementarity and sampling effects may not be mutually exclusive (Loreau *et al*., 2001), complicating our understanding of the underlying mechanisms.

The analysis here also provides qualified evidence of a positive effect of SR on the stability of yield over time, contributing to evidence of the role of biodiversity in regulating aggregate community properties (e.g. Cottingham *et al*., 2001; Worm *et al*., 2006). Analysis focused on freshwater systems in Africa demonstrates that the stability of yield decreases as SR decreases (Fig. 3). These results add to the evidence from previous, smaller‐scale studies that suggest that increased fish SR can lead to an increase in productivity and the stability of yields (Franssen *et al*., 2011; Cardinale *et al*., 2012). In particular this study suggests that findings from studies of sockeye salmon in Alaska, which show that diversity in the life history of populations increases productivity and buffers population fluctuations, particularly over long time periods (Greene *et al*., 2010), may also apply to the diversity of fish species. However, these findings do not extend to data covering Europe or to aggregate data across all African and European countries. European freshwater systems have been heavily degraded and suffered dramatic changes and species extirpations (Freyhof & Brooks, 2011); such changes may be the reason for the lack of a relationship between richness and variability of yield observed for Europe, which in turn drives the aggregate pattern across all countries.

The contribution of fish SR (despite a frequent reliance on a limited number of targeted harvest species) to yield and stability of yield (in Africa) in this study highlights the likely importance of non‐exploited species in freshwater systems globally. This is probably due to a number of functional processes carried out by species not directly harvested for consumption – such as nutrient cycling, habitat creation, water filtration and their role in the trophic web – all of which work to support the harvested species (Hensel & Silliman, 2013). Although the conclusions drawn here are based on fish SR, it is very likely that not just fish but also other components of freshwater biodiversity are important for fisheries. Unfortunately, it is not possible to disentangle the effects of fish SR and overall freshwater SR in this study due to the extremely high collinearity (88%) between these two variables [see Appendix S4 for detailed methods and results for additional analyses based on overall freshwater SR (fish, odonates, molluscs, decapods)].

The very good explanatory power of the global model (*R* ^2^ = 0.77) indicates that the results for biodiversity are very unlikely to be an artefact of another macroscale driver not considered in these analyses, and the residual variation of the model at the country level does not show any striking spatial pattern (Fig. S2). However, as with any large‐scale analysis of existing datasets, the findings of the current study are dependent on both the completeness and the accuracy of the data underpinning it, and the findings come with a number of important caveats. Firstly, there are no primary datasets for two key drivers of fishery yields (fishing effort and freshwater productivity), meaning that proxy measures which may be imperfect representations of such drivers were utilized. It is therefore possible that some of the effect attributed to biodiversity is actually due to fishing effort or productivity.

Analysis of the variability in fisheries yield over time could also be influenced by a range of factors for which there are limited data. Principal amongst these is a lack of data on variation in fishing effort, which may vary in order to stabilize catches through time. In addition, there is no way to differentiate between types of, or scales of, fisheries; indeed, subsistence catches are vastly unreported (Béné *et al*., 2007), which may in part explain the high unexplained variance in these analyses. European fisheries in particular may experience more intense management than their African counterparts (such as yield regulations and artificial stocking) and may be expected to provide more accurate reporting. However, differential reporting would not inflate the relationships between yield and fish SR reported in this study, as there is no reason to suspect a systematic bias of better recording of yields in countries with high than with low fish SR. If anything, biases in management and recording effort will have reduced the observed effects, as in general better management and recording of yields would be expected in countries with relatively low biodiversity (i.e. those in Europe) than in countries with high biodiversity (i.e. Africa).

There are also a number of issues with both the FAO and IUCN datasets. In many cases the FAO has had to rely on estimation or extrapolation to determine likely yield sizes. However, if only measured yield data are used for Africa (d.f. = 9), the *R* ^2^ for the effects of biodiversity on variability in yield increases from 0.16 to 0.21, suggesting that more accurate data could indicate an even stronger relationship. FAO yield data are currently only widely available at the country level but it would be beneficial to examine the relationships discussed here at multiple scales (e.g. catchment and subcatchment levels). Matching catchments to fisheries yields will facilitate the exploration of the link between the health of the river system and the productivity and variability of the yield in further detail. Examining the relationship at a finer resolution would also help to elucidate the role of fish SR versus the SR of other freshwater species, as the diversity of the taxonomic groups is not found to correlate at the smaller catchment scale (Darwall *et al*., 2011). Although the IUCN data are the most comprehensive freshwater data available, and indeed could be used for analyses at a finer resolution than country level, they do not provide complete global coverage because they omit important fishing regions such as China and South America.

The findings, but also the limitations, of this study have major management implications for freshwater ecosystems, for three main reasons. Firstly, as the countries with the most important inland capture fisheries also generally have the highest freshwater biodiversity, it is clear that management of these key fisheries must be sustainable in terms of both yield and conservation. This study therefore provides strong support for efforts to promote multifunctional watersheds, with a focus on sustainable fisheries management and fish conservation initiatives (Dudgeon, 2010; Cowx & Portocarrero Aya, 2011). Secondly, results suggest that fish diversity may deliver benefits for human wellbeing – particularly in terms of maintaining constant yields over time. Capture fisheries are a critical part of food security and livelihoods, particularly in developing countries, where fisheries provide a major source of protein and micronutrients, and where they are used as a safety net in times of hardship, such as due to crop failure (Béné *et al*., 2007; Dugan *et al*., 2010). As such, these results provide a powerful argument for placing biodiversity conservation centrally within fisheries management. Finally, this study makes it clear that there is a paucity of data for freshwaters, including a thorough understanding of species compositions and distributions worldwide, and for major ecosystem‐specific macroecological drivers such as productivity measures. Equally, a concentrated effort is required to increase reporting not only of inland fishery yields, but also of fishing efforts (see De Graaf *et al*., 2012). Only by doing this will we be able to fully understand the extent of the role that biodiversity plays in underpinning inland fisheries.

Inland waters are the most threatened systems globally, with dams, water extraction, pollution and invasive species recognized as some of the biggest threats to freshwater systems and to fisheries, as well as overharvesting of the fisheries themselves (Dudgeon *et al*., 2006). It is imperative that the relationships explored here should be considered within freshwater and fisheries management; the protection and conservation of species diversity in freshwater systems is a win–win outcome for human food delivery and conservation efforts to preserve freshwater ecosystems.

Biosketch This study was completed as part of the PhD thesis of **Emma Brooks**. It is based on a collaboration between the IUCN Global Species Programme's Freshwater Biodiversity Unit, which aims to provide the factual basis for conserving and managing freshwater species and support decisions for the benefit of ecosystems and human wellbeing, and the University of Southampton's Centre for Biological Sciences, a centre for excellence in spatial ecology and the understanding of ecosystem services and biodiversity.

## Supporting information


**Appendix S1** Controlling for effort in yield.
**Appendix S2** Principal components analysis of climatic variables.
**Appendix S3** Variance in yield over time.
**Appendix S4** Relationship with species richness considering multiple groups.
**Appendix S5** Supplementary figures.
**Appendix S6** Supplementary tables.
