# Supplementary material for: Global evidence of positive impacts of freshwater biodiversity on fishery yields
Source: Glob Ecol Biogeogr. 2016 Feb 1;25(5):553–62. doi: 10.1111/geb.12435 (PMC4984834; doi:10.1111/geb.12435)
Supplement: Supplementary file 1 — Appendix S1 Controlling for effort in yield. Appendix S2 Principal components analysis of climatic variables. Appendix S3 Variance in yield over time. Appendix S4 Relationship with species richness considering multiple groups. Appendix S5 Supplementary figures. Appendix S6 Supplementary tables. [file GEB-25-553-s001.docx]

### Supporting Information

#### Appendix S1 Controlling for effort in Yield

Yield is a factor not only of fisheries productivity but also of human effort and demand. This is often controlled for by either reporting yield per unit area or yield per unit effort (Kantoussan *et al*. 2014). No per unit effort or number of fishers data is comprehensively available at the country level for inland fisheries. As a proxy measure for fisheries effort we used the sum of the population living within 10km of inland waterbodies for each country. The size of the population surrounding the waterbody would be expected to reflect the reliance upon the waterbody, and thus act as a proxy for fishing effort. In an area with a limited population, yield would be expected to be lower regardless of the amount of fish potentially available.

To test the efficacy of waterside population as a proxy for fishing effort, population within a 5, 10 and 20km buffer (all log transformed) of 28 African lakes for which the number of fisher men and women could be collated (Henderson & Welcomme, 1974; Vanden Bossche & Bernacsek, 1990; Mölsä et al., 1999; van Zwieten & Njaya, 2003; Weyl, 2003; FAO, 2007; Weyl et al., 2010; Marshall & Mkumbo, 2011) was extracted. Population was extracted from a raster layer of the 2000 global rural population reported by FAO at the 5 arc-minute resolution (Salvatorre et al. 2005) using a buffer around each of the sample lakes. The 10km buffer was found to have the strongest correlation with the number of fishers (Pearson’s *r* = 0.75, *P*<0.0001), compared to the population within a 5km buffer correlation (Pearson’s *r* = 0.74, *P*<0.0001) or a 20km buffer (Pearson’s *r* = 0.70, *P*<0.0001). The analysis focused on Africa as: (a) collating fisher data on all lakes globally was not tractable and; (b) Africa has almost half (49) of the countries examined in this study and is a region where inland fisheries are known to be particularly important for rural livelihoods (Dugan *et al*. 2010).

Yield per unit area was not used in these analyses as the surface area of a country’s inland waters does not reflect the yield relationship at the country-level; for instance Denmark and Bangladesh contain a similar area of water, yet the yield for Bangladesh far exceeds that of Denmark (Fig. S1A). Fig. S1B shows that population far better correlates with the differences in yield between these two countries. Furthermore, yield per unit area is not regarded as a robust way of controlling for area in this type of ecological analysis (García-Berthou 2001).

Neither surface area nor waterside population are independent as they correlate with each other (Fig. S1C). Similarly, fish species richness also correlates with area (Fig. S1D). There are therefore multiple confounding factors, which are controlled for by including all of them as predictor variables within the multiple regression models. The covariation between variables is likely to reduce the expected size of independent effects, and therefore it is of particular interest to compare the relative importance and effect sizes between variables.

#### Appendix S2 Principal components analysis of climatic variables

It is necessary to control for productivity in the system; however no spatial data of global freshwater productivity currently exists. A surrogate for ambient and productive energy in the system – the two are highly correlated - can be achieved using climatic variables, including water metrics (i.e. precipitation) and seasonality (Hawkins et al., 2003). Climatic variables were derived by performing a principal components analysis (PCA) on a range of climatic data layers. Use of a PCA reduces multidimensionality and eliminates collinearity between variables. Nineteen spatial climatic data layers were accessed from www.worldclim.org, including mean and seasonality for temperature and precipitation variables (Hijmans et al., 2005). Mean values for each of the 19 data layers available were extracted at the country level and used within the PCA (see Table S1). A broken stick model was used as a stopping rule in order to avoid under- or over-estimating the influence of data by including the correct number of non-trivial components (Jackson 1993). These newly derived components were then included as climatic variables within the models. The broken stick stopping model resulted in the retention of the first two principal components for inclusion in further analysis. The eigenvalue of component 1 equalled 10.6, explaining 55.7% of the variance in the data, and component 2 had an eigenvalue of 4.6, explaining 24.1% of the variance. The correlations of the components with each climatic variable are shown in Table S1.

#### Appendix S3 Variance in yield over time

When examining the link between biodiversity and variation in fisheries yield, CV would not differentiate a difference between a yield which is steadily increasing or decreasing and one which is unstable but fluctuating in similar increments around the mean (see Fig. S3). Therefore a variation metric (V) has been adapted which uses differences of year on year yield to calculate the variability of yield over time within the system, where x_i_ is fisheries yield for a given year and n is the number of years within the study period:

$$V=\frac{\sqrt{\sum\left( x_{i+1}-x_{i} \right)^{2}}}{n-1}\div\overline{x}$$

Total fishing yield was extracted per country for each of the years 1981-2010. Records prior to 1981 were excluded due to the higher chance of inaccuracies and extrapolated figures with older data. The variability of yield (t) was calculated for each country for decadal increments from the years 1981 – 2010, and the mean was compared with linear regression to fish SR per country. As before, Box-Cox methodology was used to determine the most appropriate transformation. Analysis was repeated for country data subset by continent for comprehensive datasets; Africa and Europe. A comparison of CV and adjusted variability (V) results is shown in Table S5.

#### Appendix S4 Relationship with species richness considering multiple groups

A subset of the main analyses detailed in the main text (Fig. 2, Table 1and Table S2) were repeated to incorporate the species richness of other freshwater taxonomic groups. Correlations between spatial patterns of fish SR and SR of the other freshwater faunal groups at the country level were examined using Spearman’s Rho due to non-normality of the data, with corrected degrees of freedom calculated using Dutilleul’s modified test to account for spatial autocorrelation.

When SR was expanded to include odonates, molluscs and decapods, results were largely concordant with the fish only results, with a 1.35-1.45 increase in AICc (Fig. S4, Table S3-Table S4). The strength of relationship between CV and multiple freshwater taxa species richness is equal to that with fish species richness (Fig. S5). However, in both of these analyses it is not possible to disentangle the effect of overall SR from that of fish richness as there is a strong correlation between fish SR and SR of the other freshwater faunal groups at the country level ( *r_17.15_* = 0.88, *P* < 0.0001).

#### Appendix S5 Supplementary Figures


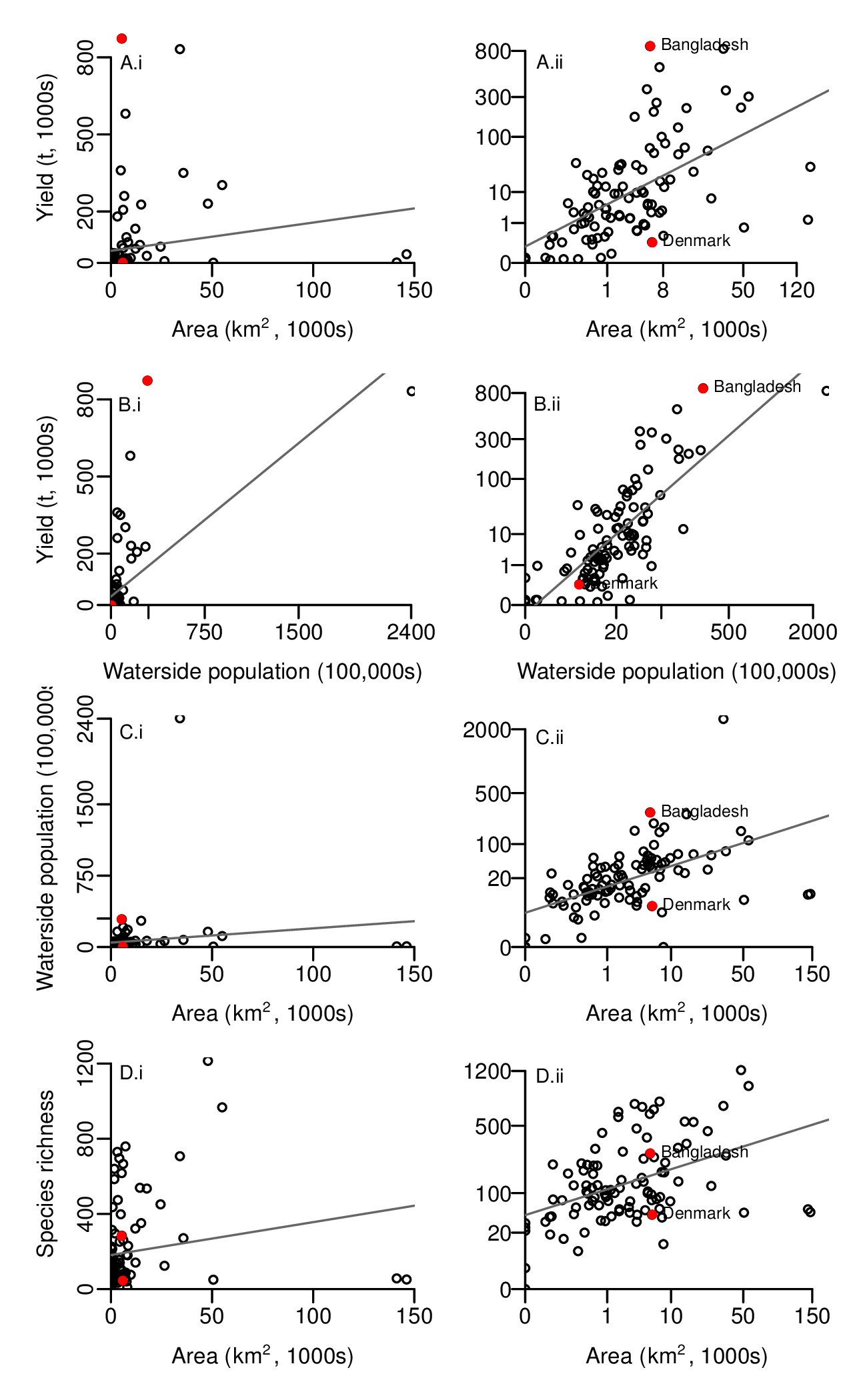


Fig. S1 Relationship between confounding model variables at the country level (N=100), (i) raw data and (ii) transformed data: A) Yield ~ Inland water surface area (cor = 0.59, *P*<0.0001); B) Yield ~ Mean population (cor = 0.70, *P*<0.0001); C) Inland water surface area ~ Mean population (cor = 0.53, *P*<0.0001), and; D) Area ~ Fish species richness (cor = 0.44, *P*<0.0001). Yield, area and population are quarter root transformed, fish species richness is cubic root transformed. Red dots indicate data from Bangladesh and Denmark for comparison.


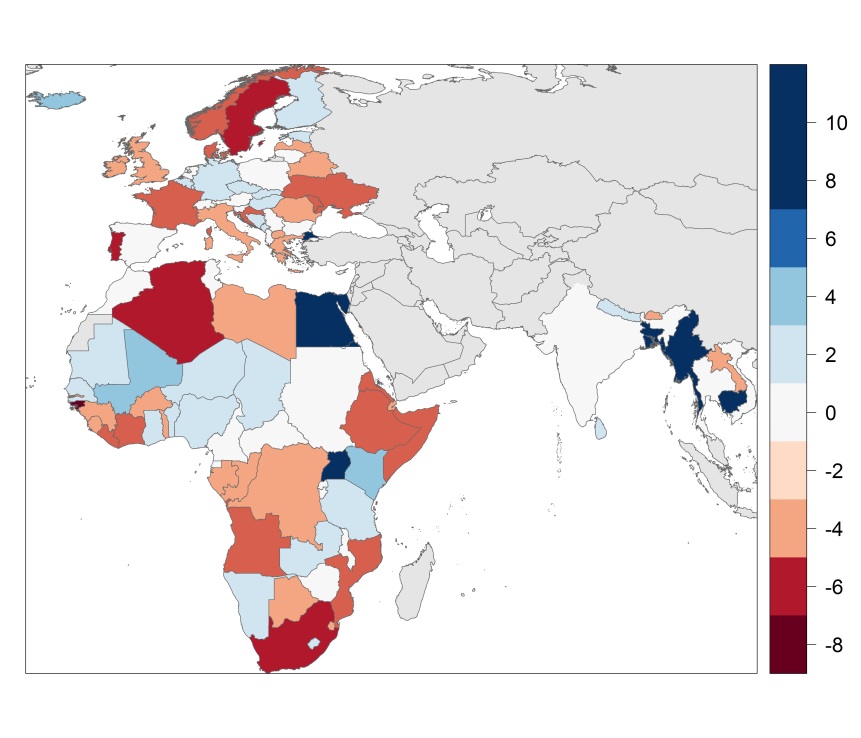


Fig. S2 Residual difference between the observed and expected values from the full spatial simultaneous autoregressive model predicting inland water capture fishery yields per country. Blue countries indicate where FAO reported yield is higher than expected from the model, red countries are where yield is lower than expected. Full model as calculated in SAR_err_: $\sqrt[4]{Yield}= \sqrt[3]{SR}+\sqrt[4]{P}+C1+C2+\sqrt[4]{A}+\sqrt[3]{E}$. SR = species richness of fishes, P = human population living within 10km of inland waterbodies, C1 = first principle component of climatic variables, C2 = second principle component of climatic variables, A = inland water area in km^2^, E = mean elevation (m).


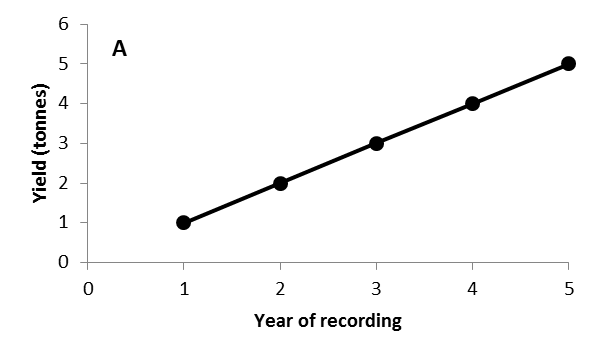

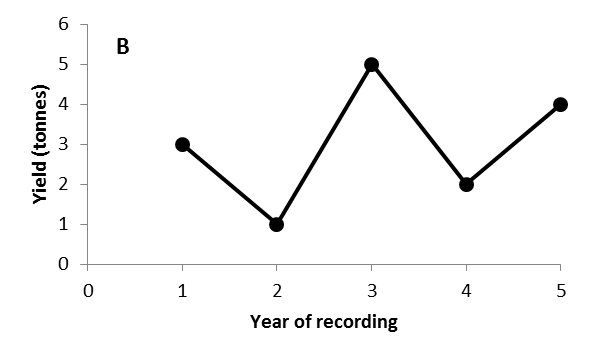


Fig. S3 Hypothetical fluctuations in yield showing that a steady increase in yield over time has the same coefficient of variation as a yield with more fluctuations, but a lower variability in year-to-year differences (V). The mean and CV for A and B are the same at 3 and 0.83 respectively. The adapted variability measure (V) is greater in B (V = 0.48) than in A (V = 0.17).


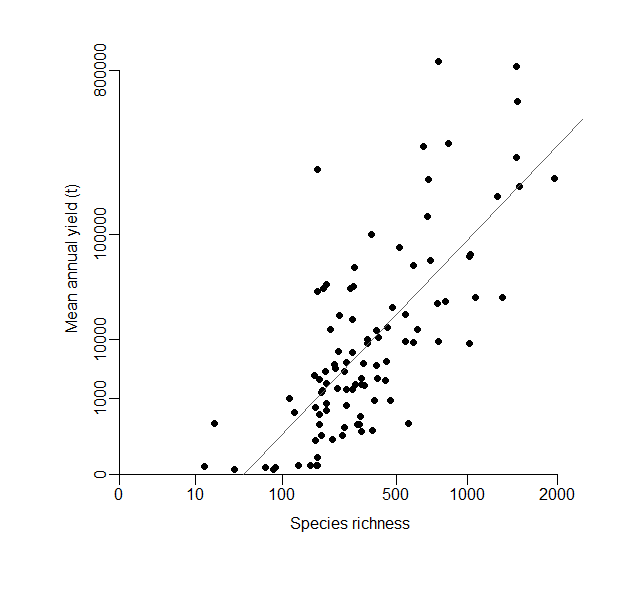


Fig. S4 Relationship between inland water capture fisheries yield (t) (axis quarter root transformed) and freshwater species richness (axis cubic root transformed), *R^2^*=0.54, F= 116.8, df=98, *P*<0.001.


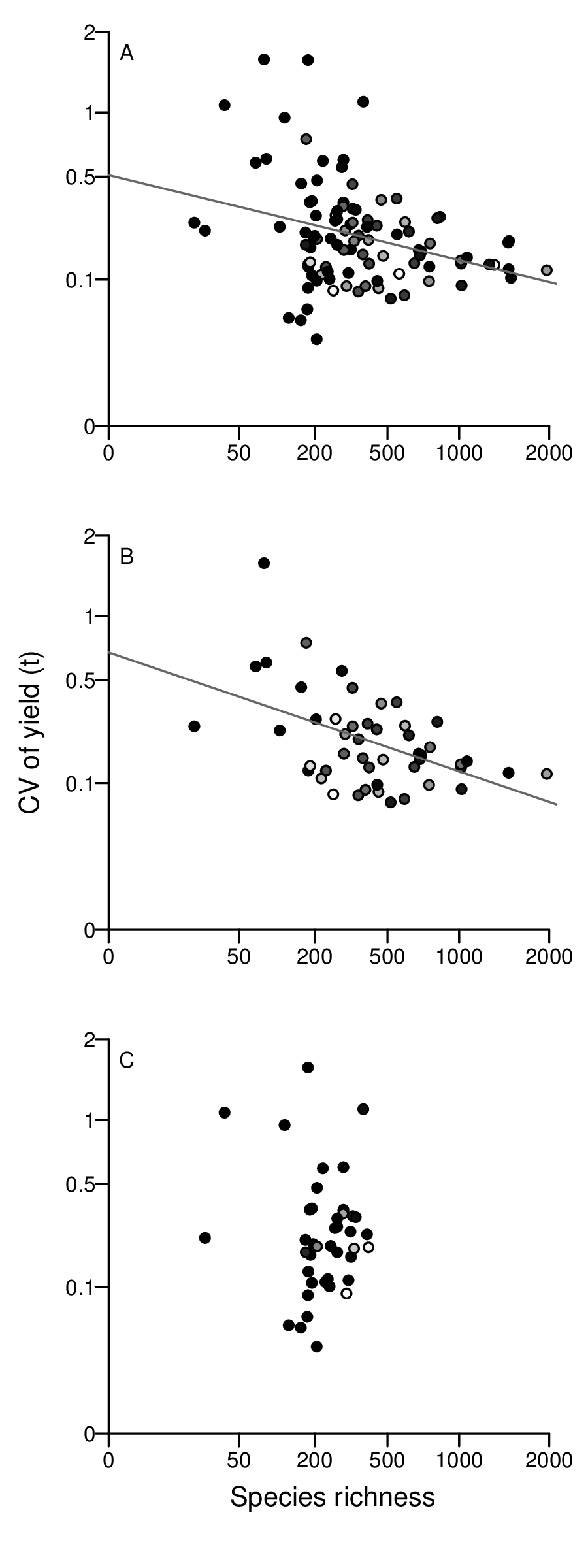


Fig. S5 Relationship between freshwater species richness and mean coefficient of variation of yield (kg) per capita (both cubic root transformed). A) All countries within boundaries of this study, *R^2^*=0.03, *F*=3.66, *df*=98, *P*=0.06. B) African countries, *R^2^*=0.16, *F*=9.68, *df*=46, *P*=0.003. C) European countries, *R^2^*=-0.03, *F*=0.01, *df*=39, *P*=0.92. Proportion of FAO country data that has been estimated or extrapolated by FAO is graded from white (all years estimated) to black (all actual data).

#### Appendix S6 Supplementary Tables

Table S1 Principal Component Analysis of country climatic variables. The first two PCA axes explained 56% and 24% of the total variability in climate conditions, respectively, and were retained as predictors for inland water fisheries yield models**.**

| **Variable** | **Comp 1** | **Comp 2** | **Comp 3** | **Comp 4** | **Comp 5** |
| --- | --- | --- | --- | --- | --- |
| Annual mean temp. | 0.98 | -0.09 | -0.09 | 0.03 | 0.13 |
| Mean diurnal range* | 0.65 | -0.56 | 0.08 | 0.04 | -0.08 |
| Isothermality† | 0.87 | 0.20 | -0.20 | -0.27 | -0.09 |
| Temp. seasonality‡ | -0.85 | -0.37 | 0.20 | 0.29 | 0.11 |
| Max temp. of warmest month | 0.86 | -0.40 | -0.06 | 0.23 | 0.19 |
| Min temp. of coldest month | 0.95 | 0.14 | -0.23 | -0.08 | 0.09 |
| Temp. annual range§ | -0.56 | -0.66 | 0.29 | 0.38 | 0.07 |
| Mean temp. of wettest quarter | 0.87 | -0.13 | 0.20 | -0.02 | 0.24 |
| Mean temp. of driest quarter | 0.89 | -0.08 | -0.30 | 0.09 | 0.01 |
| Mean temp. of warmest quarter | 0.89 | -0.32 | -0.05 | 0.20 | 0.23 |
| Mean temp. of coldest quarter | 0.98 | 0.05 | -0.14 | -0.06 | 0.05 |
| Annual precipitation | 0.29 | 0.93 | 0.13 | 0.15 | 0.02 |
| Precip. of wettest month | 0.56 | 0.72 | 0.32 | 0.20 | -0.06 |
| Precip. of driest month | -0.77 | 0.43 | -0.27 | -0.01 | 0.28 |
| Precip. seasonality\|\| | 0.84 | -0.25 | 0.28 | 0.03 | -0.11 |
| Precip. of wettest quarter | 0.53 | 0.73 | 0.34 | 0.22 | -0.07 |
| Precip. of driest quarter | -0.69 | 0.55 | -0.30 | -0.01 | 0.30 |
| Precip. of warmest quarter | 0.09 | 0.72 | 0.58 | -0.16 | 0.16 |
| Precip. of coldest quarter | 0.06 | 0.59 | -0.54 | 0.50 | -0.19 |
| Variability explained | 55.73% | 24.07% | 7.81% | 4.26% | 2.40% |

* Mean diurnal range = (Mean of monthly (max temp-min temp))

† Isothermality = Mean diurnal range/Temp annual range*100

‡ Temp seasonality = Standard deviation*100

§Temp annual range = Max temp of warmest month-min temp of coldest month

|| Precip. seasonality = coefficient of variation

Table S2 Multimodel averaged parameter estimates of top model set of SAR models of country-level inland water fisheries yield (t). Abbreviations as in Table 1.

| Parameter | Estimate | SE | z | 95% Lower CI | 95% Upper CI | Relative importance | Total effect |
| --- | --- | --- | --- | --- | --- | --- | --- |
| (Intercept) | -1.05 | 1.61 | 0.65 | -4.21 | 2.11 | NA | NA |
| SR | 1.07 | 0.32 | 3.36 | 0.45 | 1.69 | 1.00 | 1.00 |
| P | 0.13 | 0.03 | 4.12 | 0.07 | 0.18 | 1.00 | 1.00 |
| C1 | 0.40 | 0.18 | 2.26 | 0.05 | 0.75 | 1.00 | 1.00 |
| C2 | 0.15 | 0.19 | 0.78 | -0.23 | 0.53 | 0.28 | 0.28 |
| A | 0.49 | 0.13 | 3.76 | 0.24 | 0.75 | 1.00 | 1.00 |
| E | -0.37 | 0.17 | 2.12 | -0.71 | -0.03 | 1.00 | 1.00 |

Table S3 SAR models of country-level inland water fisheries yield (t) (quarter root transformed) of the four best fitting models (95% confidence model set). Shaded cells indicate which of the biodiversity, climatic and geographic variables were included in the model. SR = species richness of freshwater taxa (cubic root transformed), P = human population living within 10km of inland waterbodies (quarter root transformed), C1 = first principle component of climatic variables, C2 = second principle component of climatic variables, A = inland water area in km^2^ (quarter root transformed), E = mean elevation (m) (cubic root transformed). ΔAICc = difference between the AICc of each model and that of the best model, Wi = Akaike weights. Full model as calculated in SAR_err_: $\sqrt[4]{Yield}= \sqrt[3]{SR}+\sqrt[4]{P}+C1+C2+\sqrt[4]{A}+\sqrt[3]{E}$

| SR | P | C1 | C2 | A | E | La | Lo | Pseudo R^2^ | AIC_c_ | ΔAIC_c_ | W_i_ |
| --- | --- | --- | --- | --- | --- | --- | --- | --- | --- | --- | --- |
|  |  |  |  |  |  |  |  | 0.76 | 545.42 | 0 | 0.44 |
|  |  |  |  |  |  |  |  | 0.76 | 547.24 | 1.82 | 0.18 |

Table S4 Multimodel averaged parameter estimates of top model set of SAR models of country-level inland water fisheries yield (t). Abbreviations as in Table S3.

| Parameter | Estimate | SE | z | 95% Lower CI | 95% Upper CI | Relative importance |
| --- | --- | --- | --- | --- | --- | --- |
| (Intercept) | -3.45 | 1.85 | 1.87 | -7.07 | 0.16 | NA |
| SR | 1.13 | 0.31 | 3.60 | 0.52 | 1.75 | 1.00 |
| P | 0.12 | 0.03 | 3.73 | 0.06 | 0.18 | 1.00 |
| C1 | 0.45 | 0.16 | 2.82 | 0.14 | 0.76 | 1.00 |
| C2 | 0.15 | 0.19 | 0.80 | -0.22 | 0.53 | 0.29 |
| A | 0.52 | 0.12 | 4.24 | 0.28 | 0.77 | 1.00 |
| E | -0.37 | 0.17 | 2.18 | -0.71 | -0.04 | 1.00 |

Table S5 Comparison of variation in yield against species richness metrics. SR = Fish species richness (cube root transformed), CV = Coefficient of variation (cube root transformed), Y = Yield (t), V = Adapted variability measure (see above for details, cube root transformed). Significant relationships in bold.

| Model | All countries | | | |  | Africa | | | |  | Europe | | | |
| --- | --- | --- | --- | --- | --- | --- | --- | --- | --- | --- | --- | --- | --- | --- |
|  | *R^2^* | *F* | *df* | *P* |  | *R^2^* | *F* | *df* | *P* |  | *R^2^* | *F* | *df* | *P* |
| CV of Y ~ SR | 0.02 | 3.14 | 98 | 0.08 |  | **0.16** | **9.81** | **46** | **0.003** |  | -0.02 | 0.21 | 39 | 0.65 |
| V of Y ~ SR | **0.04** | **5.66** | **98** | **0.02** |  | **0.14** | **8.71** | **46** | **0.005** |  | -0.02 | 0.07 | 39 | 0.79 |

Table S6 Species breakdown of FAO recorded freshwater fish catch harvest per country in 2010 for the countries where this data exists. Percentage of yield (t) produced by number of species, e.g. 90.24% of Denmark’s total yield comes from just five fish species, and the total amount harvested from 10 species or fewer. Where yield has not been identified down to species level it is either categorised as grouped (e.g. identified as torpedo-shaped catfishes), or unidentified (identified as freshwater fishes not elsewhere included). Countries are ordered by yield (t), lowest to highest. Data from FAO (2011). Due to the numerical and categorical nature of the data they cannot be statistically analysed, however it is apparent that where catch has been identified down to species level, five species or fewer account for a large proportion of the yield for the majority of countries, and that instances of six or more species identified to contribute significantly to total country yield do not increase as yield (t) increases.

| **Country** | **1-5 sp.** | **6-10 sp.** | **11+ sp.** | **Grouped yield** | **Unidentified** |
| --- | --- | --- | --- | --- | --- |
| Denmark | 90.24% | 9.76% | 0.00% | 0.00% | 0.00% |
| Lesotho | 44.44% | 0.00% | 0.00% | 0.00% | 55.56% |
| Botswana | 0.00% | 0.00% | 0.00% | 98.33% | 1.67% |
| Ireland | 100.00% | 0.00% | 0.00% | 0.00% | 0.00% |
| Slovenia | 73.37% | 15.98% | 7.10% | 3.55% | 0.00% |
| Macedonia | 53.39% | 3.81% | 0.00% | 40.25% | 2.54% |
| Iceland | 100.00% | 0.00% | 0.00% | 0.00% | 0.00% |
| Latvia | 79.33% | 17.02% | 1.22% | 0.00% | 2.43% |
| Croatia | 87.72% | 10.96% | 1.32% | 0.00% | 0.00% |
| Belgium | 73.39% | 10.76% | 0.00% | 15.85% | 0.00% |
| Montenegro | 44.01% | 0.00% | 0.00% | 40.45% | 15.54% |
| Norway | 100.00% | 0.00% | 0.00% | 0.00% | 0.00% |
| Belarus | 62.21% | 12.37% | 0.22% | 25.08% | 0.11% |
| Greece | 68.12% | 4.13% | 0.22% | 8.27% | 19.26% |
| Bulgaria | 89.20% | 6.46% | 4.34% | 0.00% | 0.00% |
| Sweden | 84.21% | 15.79% | 0.00% | 0.00% | 0.00% |
| Lithuania | 79.92% | 12.37% | 4.35% | 2.90% | 0.46% |
| Slovakia | 85.01% | 6.90% | 2.18% | 5.91% | 0.00% |
| Switzerland | 31.40% | 1.33% | 0.00% | 67.15% | 0.12% |
| Netherlands | 89.76% | 0.00% | 0.00% | 3.41% | 6.83% |
| Romania | 75.95% | 12.86% | 3.58% | 7.45% | 0.16% |
| United Kingdom | 93.93% | 0.00% | 0.00% | 6.07% | 0.00% |
| France | 53.60% | 0.00% | 0.00% | 4.40% | 42.00% |
| Estonia | 94.70% | 4.60% | 0.66% | 0.00% | 0.03% |
| Albania | 63.70% | 8.60% | 0.00% | 11.40% | 16.30% |
| Italy | 1.14% | 0.00% | 0.00% | 27.41% | 71.44% |
| Czech Republic | 91.53% | 5.19% | 2.03% | 0.00% | 1.25% |
| Gambia | 0.00% | 0.00% | 0.00% | 25.91% | 74.09% |
| Ukraine | 91.55% | 5.87% | 1.65% | 0.94% | 0.00% |
| Spain | 41.11% | 0.00% | 0.00% | 0.00% | 58.89% |
| Serbia | 47.78% | 13.19% | 4.41% | 0.00% | 34.62% |
| Togo | 0.00% | 0.00% | 0.00% | 80.00% | 20.00% |
| Hungary | 70.01% | 6.11% | 0.00% | 17.91% | 5.97% |
| Morocco | 0.36% | 0.00% | 0.00% | 85.80% | 13.84% |
| Gabon | 0.00% | 0.00% | 0.00% | 49.95% | 50.05% |
| Zimbabwe | 75.24% | 0.00% | 0.00% | 9.52% | 15.24% |
| Rwanda | 100.00% | 0.00% | 0.00% | 0.00% | 0.00% |
| Burkina Faso | 0.00% | 0.00% | 0.00% | 65.43% | 34.57% |
| Germany | 5.16% | 1.48% | 0.24% | 13.15% | 79.96% |
| Burundi | 96.25% | 1.37% | 0.12% | 0.00% | 2.27% |
| Ethiopia | 18.55% | 0.00% | 0.00% | 78.29% | 3.16% |
| Poland | 9.28% | 2.99% | 1.16% | 0.00% | 86.57% |
| Benin | 3.82% | 0.00% | 0.00% | 80.38% | 15.80% |
| Finland | 82.36% | 12.96% | 2.20% | 0.00% | 2.48% |
| Laos | 0.00% | 0.00% | 0.00% | 15.86% | 84.14% |
| Senegal | 25.29% | 1.55% | 0.12% | 13.83% | 59.21% |
| Turkey | 74.76% | 4.50% | 0.25% | 19.88% | 0.61% |
| Niger | 19.25% | 0.00% | 0.00% | 65.75% | 15.00% |
| Mozambique | 28.84% | 0.00% | 0.00% | 0.00% | 71.16% |
| Sri Lanka | 0.00% | 0.00% | 0.00% | 53.90% | 46.10% |
| Sudan | 62.00% | 0.00% | 0.00% | 0.00% | 38.00% |
| Zambia | 10.24% | 0.00% | 0.00% | 0.00% | 89.76% |
| Malawi | 0.00% | 0.00% | 0.00% | 95.29% | 4.71% |
| Mali | 62.15% | 0.00% | 0.00% | 19.00% | 18.85% |
| Kenya | 85.26% | 0.00% | 0.00% | 12.10% | 2.64% |
| Thailand | 55.56% | 0.30% | 0.00% | 9.96% | 34.19% |
| Egypt | 77.85% | 1.16% | 0.00% | 15.14% | 5.84% |
| Tanzania | 70.91% | 0.00% | 0.00% | 23.61% | 5.48% |
| Nigeria | 27.82% | 1.17% | 0.00% | 67.38% | 3.63% |
| Uganda | 44.47% | 0.00% | 0.00% | 54.83% | 0.70% |
| Bangladesh | 11.70% | 0.00% | 0.00% | 0.00% | 88.30% |
| India | 0.62% | 0.00% | 0.00% | 62.68% | 36.71% |
